# SIRPα-αCD123 fusion antibodies targeting CD123 in conjunction with CD47 blockade enhance the clearance of AML-initiating cells

**DOI:** 10.1186/s13045-021-01163-6

**Published:** 2021-09-27

**Authors:** Siret Tahk, Binje Vick, Björn Hiller, Saskia Schmitt, Anetta Marcinek, Enrico D. Perini, Alexandra Leutbecher, Christian Augsberger, Anna Reischer, Benjamin Tast, Andreas Humpe, Irmela Jeremias, Marion Subklewe, Nadja C. Fenn, Karl-Peter Hopfner

**Affiliations:** 1 Gene Center and Department of Biochemistry, Ludwig-Maximilians-Universität München, Feodor-Lynen-Straße 25, 81377 Munich, Germany; 2 Research Unit Apoptosis in Hematopoietic Stem Cells, Helmholtz Zentrum München, German Research Center for Environmental Health (HMGU), Neuherberg, Germany; 3 German Cancer Consortium (DKTK), Partner Site Munich, Munich, Germany; 4 Laboratory for Translational Cancer Immunology, Gene Center, LMU Munich, Munich, Germany; 5 Department of Hematology and Oncology, Department of Medicine III, University Hospital, LMU Munich, Munich, Germany; 6 Department of Transfusion Medicine, Cellular Therapeutics and Hemostaseology, University Hospital, LMU Munich, Munich, Germany; 7 Department of Pediatrics, Dr. von Hauner Children’s Hospital, LMU Munich, Munich, Germany

**Keywords:** CD47, Acute myeloid leukaemia, CD123, Leukemic stem cells, Phagocytosis, Immunotherapy

## Abstract

**Background:**

Acute myeloid leukaemia (AML) stem cells (LSCs) cause disease relapse. The CD47 “don’t eat me signal” is upregulated on LSCs and contributes to immune evasion by inhibiting phagocytosis through interacting with myeloid-specific signal regulatory protein alpha (SIRPα). Activation of macrophages by blocking CD47 has been successful, but the ubiquitous expression of CD47 on healthy cells poses potential limitations for such therapies. In contrast, CD123 is a well-known LSC-specific surface marker utilized as a therapeutic target. Here, we report the development of SIRPα-αCD123 fusion antibodies that localize the disruption of CD47/SIRPα signalling to AML while specifically enhancing LSC clearance.

**Methods:**

SIRPα-αCD123 antibodies were generated by fusing the extracellular domain of SIRPα to an αCD123 antibody. The binding properties of the antibodies were analysed by flow cytometry and surface plasmon resonance. The functional characteristics of the fusion antibodies were determined by antibody-dependent cellular phagocytosis and antibody-dependent cellular cytotoxicity assays using primary AML patient cells. Finally, an in vivo engraftment assay was utilized to assess LSC targeting.

**Results:**

SIRPα-αCD123 fusion antibodies exhibited increased binding and preferential targeting of CD123^+^ CD47^+^ AML cells even in the presence of CD47^+^ healthy cells. Furthermore, SIRPα-αCD123 fusion antibodies confined disruption of the CD47-SIRPα axis locally to AML cells. In vitro experiments demonstrated that SIRPα-αCD123 antibodies greatly enhanced AML cell phagocytosis mediated by allogeneic and autologous macrophages. Moreover, SIRPα-αCD123 fusion antibodies efficiently targeted LSCs with in vivo engraftment potential.

**Conclusions:**

SIRPα-αCD123 antibodies combine local CD47 blockade with specific LSC targeting in a single molecule, minimize the risk of targeting healthy cells and efficiently eliminate AML LSCs. These results validate SIRPα-αCD123 antibodies as promising therapeutic interventions for AML.

**Supplementary Information:**

The online version contains supplementary material available at 10.1186/s13045-021-01163-6.

## Background

Therapeutic options for acute myeloid leukaemia (AML) are limited, and the majority of patients relapse due to persistent chemorefractory LSCs [1–3]. Targeting and eradicating the leukemic stem cell (LSC) population is therefore a prerequisite for sustained remission. CD47 is an innate immune checkpoint upregulated on LSCs, where it functions as a “don't eat me” signal by interacting with SIRPα on myeloid cells [4–6]. The first in class CD47-blocking antibody, magrolimab (Hu5F9-G4), was evaluated as a monotherapy in AML in a phase 1 trial (NCT02678338) [7, 8]. However, preclinical data support the combination of magrolimab with pro-phagocytic signals, such as activation of Fcγ receptors (FcγR) on macrophages or expression of calreticulin on target cells [8–12]. Magrolimab has consequently been combined with calreticulin-inducing azacytidine in a phase 1b trial including untreated AML patients unfit for chemotherapy and patients with intermediate to very high-risk myelodysplastic syndrome (MDS) [8, 13]. The combination demonstrated encouraging results; 64% of AML patients achieved an objective response (OR), while 56% achieved complete remission (CR) or CR with incomplete haematological recovery. In patients with high-risk MDS, 91% had an OR, and 42% had a CR (NCT03248479).

Nevertheless, CD47 is ubiquitously expressed on healthy cells as well, which generates an antigen sink lowering the effective dose and comprising a potential site of toxicity for αCD47 therapies [14, 15]. Combining the CD47 blocking domain, such as endogenous SIRPα, with a cancer-specific antibody in a single molecule can restrict the blockade of CD47 locally on antigen-expressing cells [16–18].

Similar to CD47, the interleukin-3 receptor alpha chain (CD123) is upregulated on AML LSCs and is associated with increased proliferation of AML cells and a poor prognosis [19–21]. Furthermore, high CD47 and CD123 coexpression has been demonstrated to correlate with AML chemoresistance [22]. These studies suggest that dual targeting of CD123 and CD47 could reduce the LSC count and enhance the rate and duration of response in AML patients.

To improve AML LSC targeting and clearance, we fused an αCD123 antibody with the endogenous N-terminal SIRPα immunoglobulin V-like domains and generated 1 × SIRPα-αCD123 and 2 × SIRPα-αCD123 fusion antibodies. Both of our antibodies exhibited improved binding to CD123^+^ CD47^+^ cells and stimulated efficient natural killer (NK) cell-mediated lysis of AML compared to the conventional αCD123 antibody in vitro. Importantly, SIRPα-αCD123 fusion antibodies blocked CD47 locally on CD123^+^ cells and induced phagocytosis of primary AML cells by allogeneic and autologous macrophages in vitro. Finally, the 2 × SIRPα-αCD123 antibody targeted LSCs that are capable of engrafting and reinitiating AML in an in vivo model*.*

## Materials and methods

### Expression and purification of the antibodies

αCD123 antibody light and heavy chain plasmids were generated by cloning the αCD123 variable light (V_L_) and variable heavy (V_H_) sequences of talacotuzumab [23] into the respective pFUSE2-CLIg-hK and pFUSE-CHIg-hG1 vectors (InvivoGen). For 1 × SIRPα-αCD123 and 2 × SIRPα-αCD123, one or two N-terminal SIRPα variant 1 immunoglobulin V-like domains (amino acids 31-149) were subcloned from a previously described construct [18] into the N-terminus of the αCD123 V_L_ using a (Gly_4_Ser)_4_ linker. The αCD19 V_L_ and V_H_ plasmids (clone 4G7) were cloned to generate the control molecules. The αCD47 (clone Hu5F9) V_L_ and V_H_ sequences [24] were subcloned into pFUSE2-CLIg-hK and pFUSE-CHIg-hG4, respectively. The SIRPα-Fc fusion construct (similar to TTI-621) [25] was generated by fusing the N-terminal V domain of human SIRPα variant 2 [26] to the human IgG1 Fc region of a pFUSE-CHIg-hG1 vector (InvivoGen). The plasmids were transfected into Expi293F cells (Thermo Fisher Scientific) according to the manufacturer’s protocol. After five to seven days, the supernatant was harvested, and antibodies were purified by protein A affinity chromatography followed by size exclusion chromatography using a Superdex 200 increase 10/300 GL column (GE Healthcare). Antibodies were analysed by sodium dodecyl sulphate (SDS) polyacrylamide gel electrophoresis, and stability was measured using a Tycho NT.6 (NanoTemper Technologies). The coding sequence for the CD123 extracellular domain was amplified by PCR from complementary DNA of L-428 cells and subcloned into pSecTag2/HygroC containing a His_6_-tag (Thermo Fisher Scientific). CD123 was expressed in Expi293F cells and purified by nickel affinity chromatography and size exclusion chromatography.

### Surface plasmon resonance analysis

Binding of the αCD123 antibodies to CD123 was measured using a Biacore X100 (Biacore). Antibodies were captured on a CM5 sensor chip using the Human Antibody Capture Kit (both GE Healthcare). CD123 was used at concentrations of 3.91–1000 nM, and equilibrium dissociation constants (*K*_D_) were calculated from the ratio of the rate constants (*k*_*off*_*/k*_*on*_) of the multicycle kinetics measurements using Biacore Evaluation software.

### Cell lines

All cell lines were cultured under standard conditions. MOLM-13 and Raji cells were purchased from the Deutsche Sammlung von Mikroorganismen und Zellkulturen (DSMZ). Chinese hamster ovary (CHO) cells stably overexpressing human CD47 were previously generated [18]. Expi293F cells were obtained from Thermo Fisher Scientific. Cell lines were routinely screened for mycoplasma contamination.

### Patient and healthy donor material

At initial diagnosis or relapse, AML patient samples were characterized at the Laboratory for Leukemia Diagnostics of the Klinikum der Universität München as previously described [27–29]. Peripheral blood mononuclear cells (PBMCs) were isolated from healthy donor (HD) blood or residual cells of leukoreduction chambers by Biocoll (Biochrom). RBCs were collected from HD peripheral blood. Platelet-rich plasma (PRP) was isolated from HD peripheral blood by centrifugation at 200×*g* for 20 min at 25 °C. In the binding studies, platelets were isolated from PRP in the presence of prostaglandin E1 (Merck). For patient-derived xenograft (PDX) cells, AML patient cells were serially transplanted into NOD/SCID gamma null mice (NOD.Cg-Prkdc^scid^ IL2rg^tm1Wjl^/SzJ, NSG). PDX cells were transduced with luciferase and mCherry lentiviral constructs for bioluminescence imaging [23]. For ex vivo experiments, PDX cells were grown in StemPro-34 medium with 2% FBS, L-glutamine and penicillin–streptomycin (all Gibco) supplemented with rhIL3, rhTPO, rhSCF (all Peprotech) and rhFLT3-ligand (R&D Systems). Patient characteristics are summarized in Table 1 and Additional file 1: Table S1.

**Table 1:** Patient characteristics

Patient	Age	Sex	Disease status	Karyotype	ELN genetic group	*FLT3*-ITD	*NPM1*
0276	29	F	ID	Aberrant	Adverse	wt	wt
2562	52	M	ID	Intermediate aberrant	n.a.	wt	wt
3140	74	M	ID	Normal	Intermediate	wt	wt
3073	54	M	R	Normal	Favourable	wt	wt
1233	49	F	ID	Complex aberrant	Adverse	mut	mut
3826	85	M	ID	Aberrant	Adverse	wt	wt
2449	30	F	ID	Aberrant	Favourable	wt	wt
4169	20	M	ID	Aberrant	Intermediate	wt	wt
0178	56	F	ID	Complex aberrant	Favourable	wt	wt
3386	52	M	ID	Normal	Favourable	wt	mut
3776	35	F	ID	Normal	Favourable	wt	wt
3221	59	M	ID	Normal	Favourable	wt	mut
3495	58	M	ID	Normal	Favourable	mut	wt
0885	74	F	ID	Normal	Intermediate	mut	mut
4321	50	F	ID	Normal	Intermediate	mut	mut
6789	68	M	ID	Normal	Favourable	mut	wt
0252	84	F	ID	Aberrant	Favourable	mut	mut
1421	66	F	ID	Aberrant/normal	Adverse	wt	wt
0682	56	F	ID	Complex aberrant	Adverse	wt	wt
7782	76	M	ID	Complex aberrant	Adverse	wt	wt
5964	87	F	ID	Complex aberrant	Adverse	wt	wt
AML-491 [3]	53	F	R	del(7)(q21)	Adverse	wt	wt
AML-579 [3]	51	M	R	Normal	Adverse	mut, LOH	mut
AML-640	79	M	R	t(11;15)	Intermediate	mut	mut
AML-979	56	F	R	Normal	n.a.	wt + mut subclone	mut

European LeukemiaNet (ELN), initial diagnosis (ID), relapse (R), not available (n.a.), wild type (wt), mutated (mut), loss of heterozygosity (LOH), female (F), male (M)

### Antibodies and flow cytometry

Commercial antibodies were from Biolegend (San Diego) unless otherwise stated. Human IgG1 isotype control (QA16A12) and αCD47 (B6H12, eBioscience) were used in binding, CD47 blocking and functional experiments. FITC or APC α human IgG (αhIgG, HP6017) was used for binding, and FITC αCD47 (B6H12, eBioscience) was used in CD47 blocking experiments for secondary staining. APC and FITC isotype (MOPC-21), APC αCD123 (6H6) and FITC αCD47 (B6H12) were used for surface expression analysis. Surface antigen density was evaluated using QIFIKIT (Agilent Technologies). Flow cytometry was performed using the Guava easyCyte 6HT (Merck Millipore), the Cytoflex LX (Beckman Coulter) or the BD LSRFortessa (Becton Dickinson). As a measure of antibody binding, the median fluorescence intensity (MFI) ratio was calculated by dividing the MFI of the tested antibody by the MFI of the corresponding isotype. Antibodies were considered to bind the cells if the intensity exceeded an MFI ratio of 1.5.

### Competitive binding assays

PKH26 (Sigma-Aldrich)-labelled MOLM-13 was incubated with a 20-fold excess of red blood cells (RBCs) and antibodies. APC αhIgG (HP6017) or APC αmIgG (Poly4053) was used for secondary labelling. For assays with PBMCs, calcein AM (Thermo Fisher Scientific) or CellTrace™ calcein red-orange AM (Thermo Fisher Scientific)-labelled MOLM-13 cells were incubated with a fivefold excess of PBMCs and antibodies. APC or FITC αhIgG (HP6017) was used for secondary antibody labelling.

### Platelet aggregation

PRP was centrifuged at 15,000×*g* for 2 min to obtain platelet-poor plasma (PPP). PRP was incubated in the presence of 100 nM antibodies, and absorbance was measured at 595 nm using an Infinite M100 plate reader (TECAN) for 16 min. The percentage of aggregation was calculated as $({\text{platelet aggregation}} \left[ \% \right] = 100 \times \frac{{\left( {\text{OD PRP - OD sample}} \right)}}{{\left( {\text{OD PRP - OD PPP}} \right)}})$ [30].

### Antibody-dependent cellular phagocytosis (ADCP) assay

Monocytes were enriched using a classical monocyte isolation kit (Miltenyi) and were differentiated into macrophages in the presence of 100 ng/ml MCSF (Biolegend) for 5–7 days. Macrophages were labelled with calcein AM and incubated with CellTrace™ calcein red-orange AM-labelled target cells and antibodies at 50 pM or 50 nM for 3 h at 37 °C at a 1:1 effector-to-target (E:T) ratio.

### Antibody-dependent cellular cytotoxicity (ADCC) assays

NK cells were enriched using a NK cell isolation kit (Miltenyi). MOLM-13 cells were labelled with calcein AM and incubated with NK cells and antibodies for 4 h at 37 °C at a 5:1 E:T ratio. In the competitive ADCC assay, NK cells were incubated with labelled MOLM-13 or Raji cells mixed with unlabelled Raji or MOLM-13 cells, respectively, at a 5:1:1 E:T:T ratio. Triton X-100 (2.5%, Sigma-Aldrich) was used for maximum lysis. Fluorescence intensity (FI) from calcein AM release was measured using an Infinite M100 plate reader, and lysis was calculated as (${\text{specific lysis}} \left[ \% \right] = 100 \times \frac{{{\text{FI}}\left( {\text{antibody stimulation}} \right) - {\text{FI}}\left( {{\text{untreated}}} \right)}}{{{\text{FI}}(\max ) - {\text{FI}}\left( {{\text{target}}} \right)}}$). Data were fitted to a four-parameter dose–response curve.

ADCC assays of AML patient samples were performed in α-MEM (Thermo Fisher Scientific) supplemented with 12.5% foetal calf serum, 12.5% horse serum, 1% penicillin, 1% streptomycin, 1% glutamine (Invitrogen) and a distinct cytokine cocktail on irradiated MS-5 cells in a long-term culture as described elsewhere [31, 32]. AML cells were incubated with HD NK cells and 10 nM antibodies for 20 h at 37 °C at a 5:1 E:T ratio. Dead cells were excluded as 7-AAD (BioLegend) or LIVE/DEAD™ Fixable Aqua (Thermo Fisher Scientific)-positive cells. CD33^+^ or CD123^+^ AML cells were determined by BV421, APC αhCD33 (WM53) or PE αhCD123 (6H6) labelling, respectively. Additionally, APC-Cy7 or FITC αhCD69 (FN50) and the corresponding isotype control (MOPC-21) were used to determine the percentage of CD69^+^ cells. In some experiments, NK cells were labelled with CellTrace™ CFSE or CellTrace™ Far Red (both Thermo Fisher Scientific) according to the manufacturer’s recommendations. Cell populations were assessed by flow cytometry, and the percentage of viable CD33^+^ or CD123^+^ AML cells was normalized to the human IgG1 isotype control sample. The percentage of CD69^+^ cells was normalized to the human IgG1 isotype control sample.

In the AML PDX ADCC, AML-491, AML-979, and AML-640 were incubated with NK cells and 100 nM antibodies for 20 h at 37 °C at a 5:1 E:T ratio. Cells were labelled with LIVE/DEAD Fixable Aqua, and the proportion of live mCherry^+^ cells was determined by flow cytometry and normalized to the isotype control.

### In vivo engraftment experiments

To evaluate the targeting of AML cells with leukaemia-initiating properties, ex vivo NK cell-mediated ADCC was performed using the αCD123 antibody, the 2 × SIRPα-αCD123 fusion antibody or isotype antibody as a control, and surviving cells were used in an in vivo engraftment experiment. To this end, PDXs AML-491 and AML-579 [33, 34] were incubated with HD NK cells at an E:T ratio of 5:1 and antibodies for 20 h. After ADCC, residual mCherry^+^ PDX cells were separated from NK cells and quantified by fluorescence-activated cell sorting (FACS) using a FACSAria III (BD Biosciences). According to previous data [34] and assuming that the isotype control antibody did not alter LIC frequency, we injected cell numbers corresponding to 10 leukaemia-initiating cells (10 × LIC, *n* = 5) and 100 × LIC (*n* = 5) for AML-491 or 14 × LIC (*n* = 4) and 140 × LIC (*n* = 2) for AML-579 by counting and diluting sorted cells of the isotype control suspensions. To enable comparison between the groups, equal volumes of αCD123 and 2 × SIRPα-αCD123 antibody ex vivo cell suspensions were sorted and injected intravenously into 10- to 12-week-old male (AML-491) or female (AML-579) NSG mice. Positive AML engraftment was analysed by in vivo bioluminescence imaging (BLI), and total flux was quantified as previously described [33]. Mice exhibiting a total flux greater than 5 × 10^7^ photons per second were classified as exhibiting positive engraftment; mice displaying no positive imaging signal within 28 weeks after transplantation were classified as negative for engraftment. To evaluate the percentage of human CD33^+^ cells in peripheral blood, PE anti-human CD33 (WM53, BD Biosciences) and PE isotype control (MOPC-21, BD Biosciences) were used. Mice exhibiting any clinical signs of illness or end-stage leukaemia (total flux > 2 × 10^10^ photons/s; hCD33^+^ cells in peripheral blood > 50%) were euthanized. Three mice died in narcosis during imaging and were counted as positive according to the last imaging signal or were excluded if not engrafted.

### Data analysis

Statistical evaluation was performed using GraphPad Prism versions 6.07 and 8.1.2 (GraphPad). Datasets were analysed using one-way analysis of variance (ANOVA) including a test to determine equal variances within the groups and correction for multiple testing using Holm-Sidak's test. Chi-squared test was used to determine whether there is a statistically significant difference in the growth of engrafted AML PDX cells. A Kaplan–Meier plot was generated to depict AML engraftment and survival by treatment group, and significance was assessed using the log-rank Mantel-Cox test. Extreme limiting dilution analysis was performed using the injected cell number and number of AML engrafted mice as inputs as previously described [35] (Figure 7; Additional file 1: Table S2). The results were considered statistically significant at the following values and are marked in the figures as follows: *p *value < 0.05 (*), < 0.01 (**), < 0.001 (***), < 0.0001 (****).

## Results

### Generation and characterization of SIRPα-αCD123 fusion antibodies

**Fig. 1:**
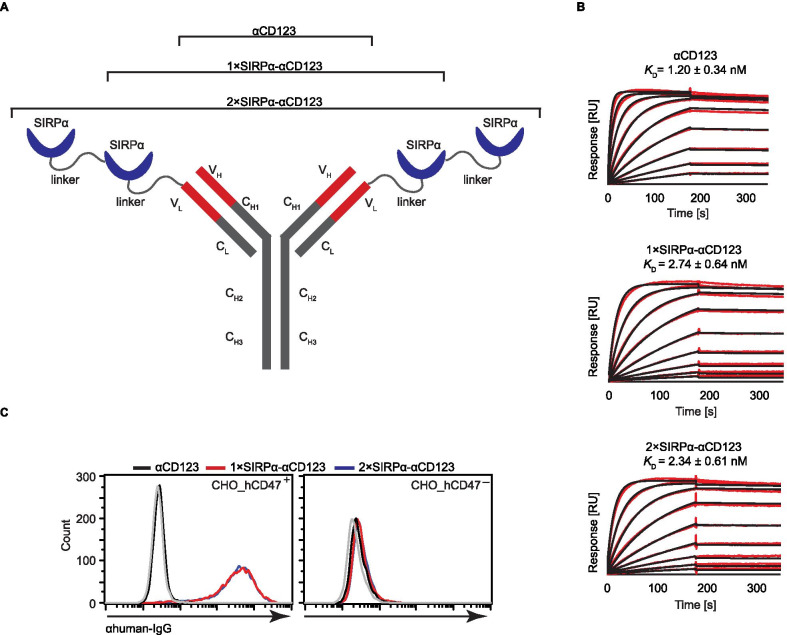
SIRPα-αCD123 fusion antibodies bind to CD123 and CD47. **A** Structure of SIRPα-αCD123 fusion antibodies. V_H_—variable heavy, C_L_—constant light, C_H1_—constant heavy 1, C_H2_—constant heavy 2, C_H3_—constant heavy 3. **B** Different CD123 concentrations binding to the antibody constructs measured using SPR. Raw data are shown in red; black curves were fitted to a 1:1 interaction. *K*_D_ values represent mean values from *n* = 3 independent experiments ± standard error of the mean (SEM). **C** Binding of antibodies to CHO_hCD47^+^ and CHO_hCD47^–^ cells at 100 nM concentration measured by flow cytometry. The grey line indicates nonspecific staining of the isotype control and secondary antibody. Histograms show 1 of 3 experiments with similar results

The 1 × SIRPα-αCD123 recombinant antibody was generated by fusing the N-terminal SIRPα immunoglobulin V-like domain to the αCD123 antibody light chain via a flexible polypeptide linker (Fig. 1A). Likewise, for 2 × SIRPα-αCD123, a second SIRPα domain was connected to the N-terminus of 1 × SIRPα-αCD123 (Fig. 1A). Antibodies were produced in Expi293F cells, purified from cell culture supernatants and analysed by size exclusion chromatography and SDS–polyacrylamide gel electrophoresis (Additional file 1: Figure S1A-B). Thermal stability was assessed by measuring changes in the intrinsic fluorescence of the proteins using Tycho NT.6 (Additional file 1: Figure S1C). To investigate whether the N-terminal fusion of the SIRPα domains alters the binding to CD123, we determined the *K*_D_ values using a Biacore assay. The *K*_D_ values were in the low nanomolar range for all constructs, indicating that the high affinity for CD123 was not affected by the fusion of the SIRPα domains (Fig. 1B). We further evaluated binding of the antibodies to CHO cells stably overexpressing human CD47 (hCD47) by flow cytometry (Table 2). As expected, 1 × SIRPα-αCD123 and 2 × SIRPα-αCD123 bound to^+^CHO_hCD47^+^ cells but not to ^–^CHO_hCD47^-^ cells (Fig. 1C). These binding experiments indicate that the αCD123 and SIRPα domains can bind to their respective targets in the fusion antibody.

**Table 2:** Antigen expression levels

Cell type	CD123	CD47	CD19
MOLM-13	13 723 ± 1 108	67 703 ± 3 784	30 ± 2
Raji	94 ± 95	170 868 ± 37 029	141 688 ± 19 997
CHO^CD47+^	104 ± 68	1 424 894 ± 329 869	n. d.
CHO^CD47−^	159 ± 50	532 ± 35	n. d.
RBC	106 ± 33	33 841 ± 2 221	n. d.

Determined using QIFIKIT. Data are shown as the means ± SEM (*n* = 2–3). Not determined (n. d.)

### SIRPα-αCD123 fusion antibodies specifically bind to CD123^+^CD47^+^ AML cells

**Fig. 2:**
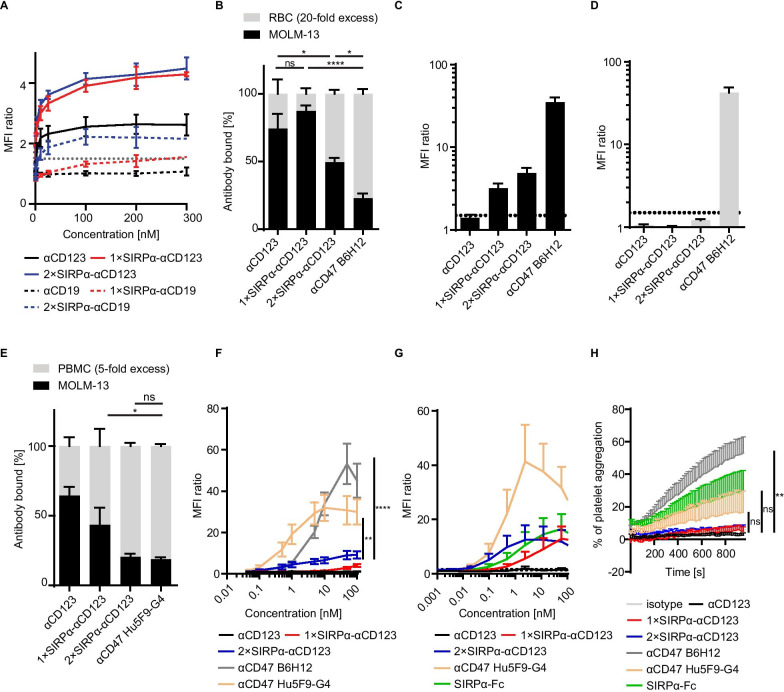
SIRPα-αCD123 fusion antibodies preferentially bind to MOLM-13 cells in the presence of RBCs. **A** Binding of SIRPα-αCD123 fusion antibodies to MOLM-13 cells assessed by flow cytometry-based MFI ratio. The dotted line indicates an MFI ratio of 1.5 as the cut-off for positivity. Shown are the mean values from *n* = 2–3 independent experiments ± SEM. **B** Percentage of 100 nM antibodies targeting MOLM-13 cells or RBCs measured by flow cytometry at a 20-fold excess of RBCs. **C** MFI ratios of antibody binding to MOLM-13 cells in the presence of a 20-fold excess of RBCs. **D** MFI ratios of antibody binding to RBCs in the presence of MOLM-13 cells. The results of independent experiments with 4 different RBC donors represented as the mean ± SEM are shown. **E** Percentage of 100 nM antibodies targeting MOLM-13 cells or PBMCs measured by flow cytometry at a fivefold excess of PBMCs. Shown are mean values from *n* = 6 donors ± SEM. **F** Binding of SIRPα-αCD123 fusion antibodies to PBMCs assessed by flow cytometry-based MFI ratio. Shown are mean values from *n* = 5 donors ± SEM. **G** Binding of SIRPα-αCD123 fusion antibodies to platelets assessed by flow cytometry-based MFI ratio. Mean values from *n* = 4 donors ± SEM are shown. **H** Platelet aggregation induced by 100 nM antibody over time. Mean values from *n* = 4 donors ± SEM are shown. **F** and **H** *p *values are the same for both SIRPα-αCD123 fusion antibodies. Statistical differences were determined by one-way ANOVA using Holm–Sidak's post hoc test. *p < 0.05, **p < 0.01, ***p < 0.001, ****p < 0.0001

Next, we used the CD123^+^ CD47^+^ AML cell line MOLM-13 in a flow cytometry-based binding assay to study the dual targeting properties of the antibody constructs (Fig. 2A, Table 2). The binding of 1 × SIRPα-αCD123 and 2 × SIRPα-αCD123 to MOLM-13 cells was stronger than that of the αCD123 antibody, indicating a contribution by the SIRPα domain. The αCD19 SIRPα fusion antibodies mediated only weak binding to CD19^–^ MOLM-13 cells due to some low binding of the SIRPα domains (Fig. 2A, Table 2). In summary, we hypothesize that the strong binding of the SIRPα-αCD123 antibodies to MOLM-13 cells is due to avidity-dependent binding to both CD123 and CD47.

The physiological interaction of the SIRPα domain and CD47 is approximately 100-fold weaker than the affinity of the αCD123 antibody for CD123 [26, 36]. Therefore, we postulated that the high affinity αCD123 drives the preferential binding of SIRPα-αCD123 fusion antibodies onto CD123^+^CD47^+^ leukemic cells over CD123¯CD47^+^ healthy cells. To test this hypothesis, we first utilized RBCs as highly abundant healthy cells expressing CD47 (Fig. 2B, Table 2).

We observed selective binding to MOLM-13 cells using the 1 × SIRPα-αCD123 antibody even in the presence of a 20-fold excess of RBCs (Fig. 2B, C). 2 × SIRPα-αCD123, on the other hand, was also detected on the surface of RBCs, indicating that the additional SIRPα domains can increase the competition between CD47^+^ MOLM-13 cells and RBCs (Fig. 2B, C). Nevertheless, the RBC targeting observed for 2 × SIRPα-αCD123 was very weak, with a binding intensity far below an MFI ratio of 1.5 (Fig. 2D). In contrast, the high affinity αCD47 B6H12 antibody demonstrated a substantial on-target off-leukaemia effect, as it primarily bound to RBCs with a high MFI ratio (Fig. 2B–D). We concluded that despite carrying the SIRPα domains, the SIRPα-αCD123 fusion antibodies target MOLM-13 cells more than the high affinity αCD47 and avoid the antigen sink generated by the RBCs.

In another set of experiments, we investigated the selective binding of our antibodies to MOLM-13 cells in the presence of HD PBMCs (Fig. 2E). From PBMCs, plasmacytoid dendritic cells express CD123 and are targeted by the αCD123 antibody CSL362 [37]. We also found that some of our αCD123 binds to PBMCs; however, the majority of the antibodies still bound to MOLM-13 cells (Fig. 2E). The 1 × SIRPα-αCD123 antibody bound PBMCs to a considerable extent, but higher selective binding to MOLM-13 cells was observed compared to the αCD47 Hu5F9-G4 clone. 2 × SIRPα-αCD123 targeted MOLM-13 cells similarly to αCD47 Hu5F9-G4. However, when we analysed binding of the antibodies to PBMCs alone, we observed that our fusion antibodies bound PBMCs significantly less than the αCD47 Hu5F9-G4 and B6H12 antibodies (Fig. 2F). These data indicate that although our fusion antibodies seem to target PBMCs more than RBCs, they bind to PBMCs to a lesser extent than the high affinity αCD47 antibodies.

In addition to binding to RBCs, CD47-targeting agents have been reported to bind platelets and interfere with their function [38, 39]. We therefore investigated whether our SIRPα-αCD123 fusion antibodies target platelets and induce their aggregation (Fig. 2G–H). Indeed, SIRPα-αCD123 fusion antibodies bound to platelets similarly to the SIRPα-Fc construct but less than the αCD47 Hu5F9-G4 control (Fig. 2G). However, SIRPα-αCD123 antibodies did not induce aggregation of platelets, unlike SIRPα-Fc, αCD47 Hu5F9-G4 and especially αCD47 B6H12 antibodies (Fig. 2H). These experiments suggest that binding of the constructs does not directly correlate with a functional effect and indicate that our SIRPα-αCD123 fusion antibodies do not stimulate platelet aggregation.

### SIRPα-αCD123 fusion antibodies block CD47 and induce phagocytosis of MOLM-13 cells in vitro

SIRPα-αCD123 fusion antibodies were designed to inhibit the CD47-SIRPα axis locally on CD123^+^ cells. To examine this, we performed a blocking assay using labelled αCD47 antibodies that interfere with the binding of SIRPα. Despite the weak affinities of the SIRPα domains, 1 × SIRPα-αCD123 and 2 × SIRPα-αCD123 were able to block CD47 molecules on MOLM-13 cells. Not surprisingly, maximum blockade was observed with the high affinity αCD47 antibody. In comparison, 1 × SIRPα-αCD123 did not block CD47 on CD123¯ Raji cells, and 2 × SIRPα-αCD123 minimally blocked CD47 (Additional file 1: Figure S2A), indicating that binding of the αCD123 moiety to target cells is required for efficient disruption of the CD47-SIRPα axis.

**Fig. 3:**
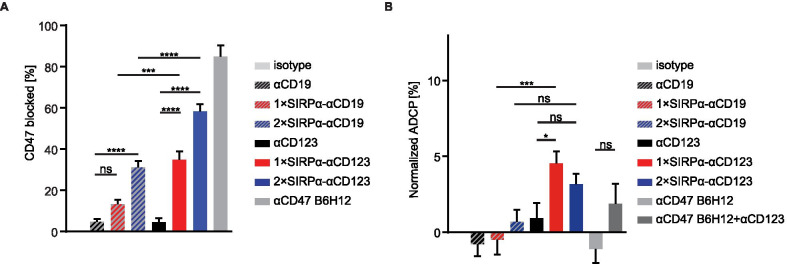
SIRPα-αCD123 fusion antibodies block CD47 and stimulate phagocytosis of MOLM-13. **A** CD47 blockade on MOLM-13 cells with 100 nM antibodies determined by FITC αCD47 binding using flow cytometry. Background fluorescence was subtracted from the FITC αCD47 signal and normalized to isotype to calculate the CD47 blockade. Mean ± SEM of *n* = 4 independent experiments. **B** ADCP of MOLM-13 cells at a 50 pM concentration of antibodies after 3 h at an E:T ratio of 1:1. ADCP was measured as the percentage of double-positive cells from macrophages and normalized to the isotype control. Bar charts represent the mean ± SEM from *n* = 7 different donors. Statistical differences were determined by one-way ANOVA using Holm-Sidak's post hoc test. *p < 0.05, ***p < 0.001, ****p < 0.0001, not significant (ns)

We next examined whether CD47 blockade with concomitant engagement of FcγRs stimulates the ADCP of MOLM-13 cells by HD-derived macrophages (Fig. 3B). Indeed, phagocytosis was significantly boosted by 1 × SIRPα-αCD123 compared to αCD123. 2 × SIRPα-αCD123 also induced elevated phagocytosis, but this was not statistically significant. In contrast, αCD47 did not stimulate phagocytosis either alone or in combination with αCD123 in this setting. The respective αCD19 controls did not have an effect on phagocytosis. In summary, SIRPα-αCD123 fusion antibodies boost ADCP in MOLM-13 cells, whereas αCD123 and αCD47 antibodies alone lack this ability.

### SIRPα-αCD123 fusion antibodies induce enhanced phagocytosis of patient-derived AML cells by allogeneic and autologous macrophages in vitro

**Fig. 4:**
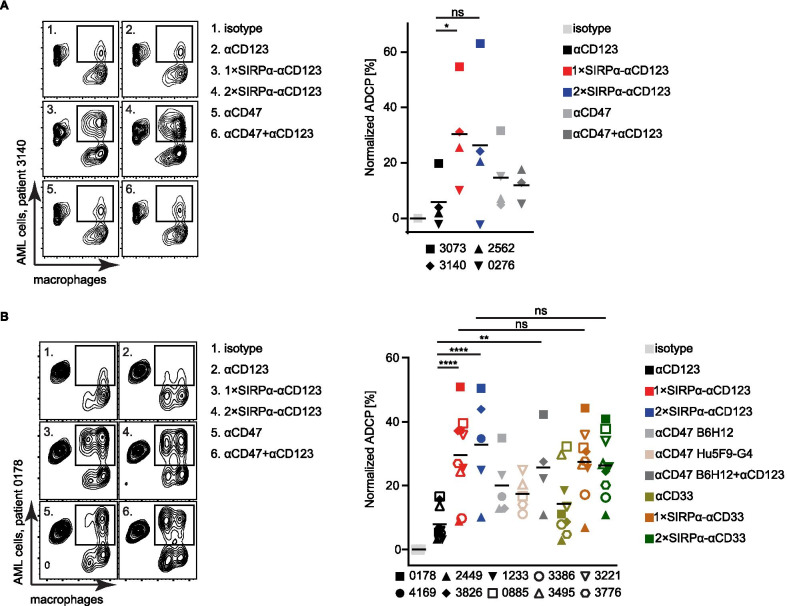
SIRPα-αCD123 fusion antibodies stimulate phagocytosis of AML patient cells by allogeneic and autologous macrophages. AML target cells and macrophages were incubated with 50 nM antibody for 3 h at an E:T ratio of 1:1. ADCP was measured as the percentage of double-positive cells (gated) from all macrophages. Each symbol represents one patient (Table 1). **A** Phagocytosis of AML patient cells by allogeneic macrophages (*n* = 4). **B** Phagocytosis of AML patient cells by autologous macrophages (*n* = 10). Statistical differences were determined by one-way ANOVA using Holm-Sidak's post hoc test. *p < 0.05, ****p < 0.0001, not significant (ns)

We further investigated the stimulation of phagocytosis by SIRPα-αCD123 antibodies using primary AML patient-derived blasts as targets and allogeneic or autologous monocyte-derived macrophages as effector cells (Fig. 4A, B). We observed enhanced overall phagocytosis by primary AML cells compared to MOLM-13 cells. Allogeneic macrophages from HDs mediated significantly higher ADCP with the 1 × SIRPα-αCD123 fusion antibody compared to αCD123. The 2 × SIRPα-αCD123 had a similar effect (Fig. 4A). More importantly, these results were confirmed in the autologous setting (Fig. 4B). Phagocytosis mediated by 1 × SIRPα-αCD123 and 2 × SIRPα-CD123 was significantly higher than that mediated by αCD123. αCD47 antibodies B6H12 and Hu5F9-G4 alone or in combination with αCD123 antibody induced similar ADCP as SIRPα-αCD123 fusion antibodies. When comparing SIRPα-CD123 fusion antibodies to similar αCD33-based constructs [18], we did not observe significant differences in the ability to induce phagocytosis of AML cells (Fig. 4B). Taken together, these data reveal that SIRPα-αCD123 fusion antibodies represent an effective tool to overcome the CD47-mediated inhibition of phagocytosis in AML.

### SIRPα-αCD123 fusion antibodies induce NK cell-mediated specific lysis of AML cells in vitro

**Fig. 5:**
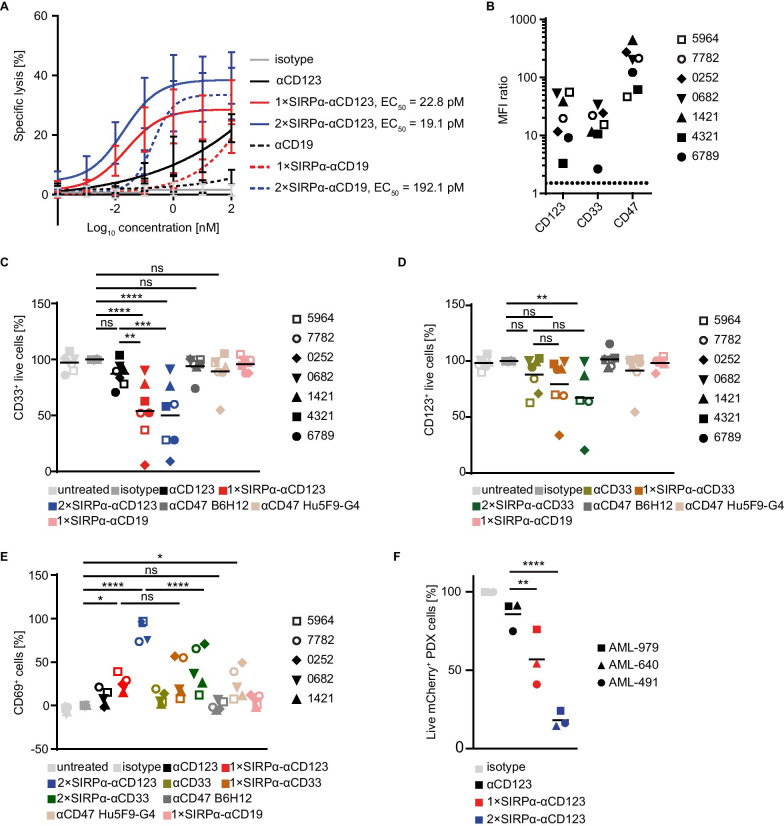
SIRPα-αCD123 fusion antibodies enhance NK cell-mediated lysis of MOLM-13 and PDX AML cells. **A** NK cell-mediated dose-dependent lysis of MOLM-13 cells after 4 h at an E:T ratio of 5:1 measured by calcein AM release. Mean values ± SEM for *n* = 6 different NK cell donors are shown. EC_50_ values were calculated where possible. **B** Expression of CD123, CD33 and CD47 in primary AML samples assessed by flow cytometry. **C**, **D** NK cell-mediated lysis of AML cells in long-term culture at a 10 nM antibody concentration after 20 h at an E:T ratio of 5:1 measured by flow cytometry. The results from *n* = 7 different AML patient samples are represented as different symbols, and their mean values are shown. **E** Percentage of CD69^+^ cells measured by flow cytometry. **F** NK cell-mediated lysis of PDX cells at 100 nM antibody concentration after 20 h at an E:T ratio of 5:1 measured by flow cytometry. The results from *n* = 3 different AML patient samples are represented as different symbols, and their mean values are shown. Statistical differences were determined by one-way ANOVA using Holm-Sidak's post hoc test. *p < 0.05, **p < 0.01, ***p < 0.001, ****p < 0.0001

ADCC by NK cells is one of the primary mechanisms by which IgG1 antibodies induce the elimination of antibody-bound cells in addition to macrophage-mediated ADCP [40]. Therefore, we analysed specific lysis of MOLM-13 cells by HD-derived NK cells (Fig. 5A). αCD123 induced moderate dose-dependent lysis of MOLM-13, whereas 1 × SIRPα-αCD123 and 2 × SIRPα-αCD123 were more potent. We postulated that SIRPα-αCD123 fusion antibodies are more efficient due to the avidity-dependent targeting of both CD123 and CD47. The respective αCD19 controls induced lysis of MOLM-13 cells only at high concentrations, which can be attributed to autonomous targeting of CD47 by the fused SIRPα domain. Nevertheless, the half maximal effective concentration (EC_50_) was considerably lower for 2 × SIRPα-αCD123 (19.1 pM) than for the 2 × SIRPα αCD19 analogue (192.1 pM), demonstrating target antigen-specific cytotoxicity. This was further demonstrated in a competitive ADCC assay in which CD123^+^ MOLM-13 cells were mixed with CD123¯ Raji cells (Additional file 1: Figure S2B). In this setting, Raji cells were not lysed by 1 × SIRPα-αCD123 and 2 × SIRPα-αCD123 only exerted an effect at high concentrations. In summary, although independent binding of the SIRPα domains can cause some lysis of target cells at high concentrations, we consider high affinity αCD123 binding to be a prerequisite for targeting by SIRPα-αCD123 fusion antibodies.

The ability of SIRPα-αCD123 fusion antibodies to activate NK cells was further investigated using AML patient cells. First, we used blasts from primary AML patients (Fig. 5B) in a long-term culture system with HD-derived NK cells as effectors [31]. Compared to isotype controls and αCD123, 1 × SIRPα-αCD123 and 2 × SIRPα-αCD123 antibodies significantly boosted the cytotoxicity by NK cells, leading to reduced numbers of AML cells (Fig. 5C). As expected, the αCD47 antibodies B6H12 and Hu5F9-G4 and the 1 × SIRPα αCD19 control molecule did not stimulate lysis of AML cells (Fig. 5C). From the αCD33 constructs, only the 2 × SIRPα αCD33 analogue induced significant lysis of AML cells compared to the isotype control (Fig. 5D). When analysing the NK cell population of the ADCC samples, we observed a significant upregulation of the activation marker CD69 with 1 ×- and 2 × SIRPα-αCD123 (Fig. 5E). Treatment with 2 × SIRPα-αCD123 induced especially potent CD69 upregulation, which was also significantly greater than that induced by the 2 × SIRPα αCD33 analogue (Fig. 5E). Interestingly, the αCD47 antibody Hu5F9-G4 induced slight upregulation of CD69 (Fig. 5E). Together, these results demonstrate that in addition to highly effective FcγR-dependent ADCC stimulation, SIRPα-αCD123 antibodies might further activate NK cells via mechanisms related to CD47 blockade.

Next, we used AML PDX cells as target cells. Here, we observed that 1 × SIRPα-αCD123 and 2 × SIRPα-αCD123 both dramatically increased the lysis of AML PDX cells compared to αCD123 (Fig. 5E). This again highlights that our fusion antibodies enhance NK cell-mediated lysis of patient-derived AML cells.

### SIRPα-αCD123 fusion antibodies have the potential to target AML stem cells

**Fig. 6:**
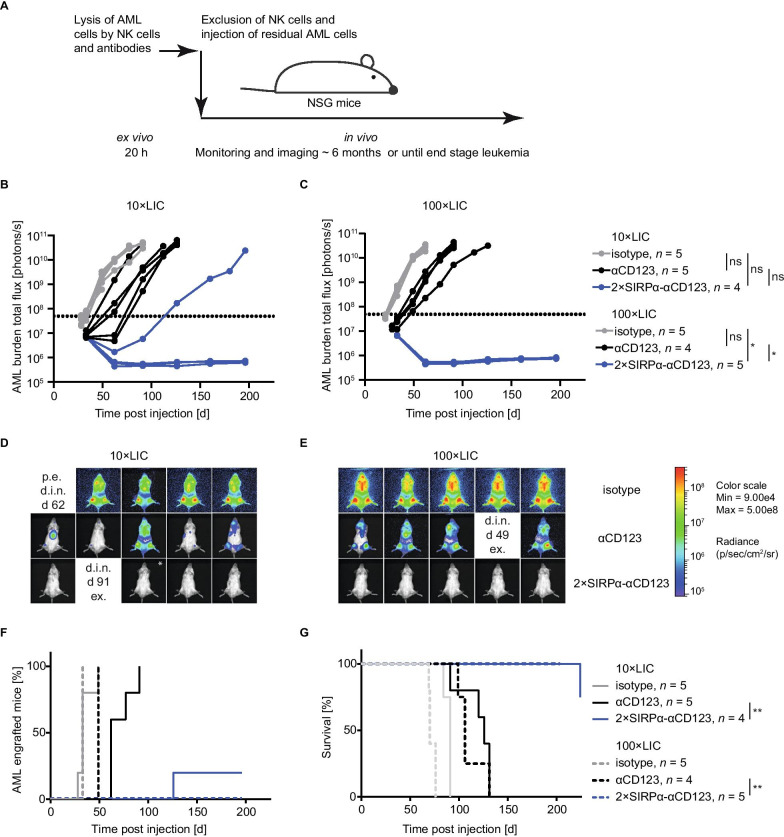
Ex vivo treatment with 2 × SIRPα-αCD123 prevents outgrowth of AML-491 PDX cells in vivo. **A** Experimental design of the engraftment assay. Residual AML cell from ADCC in (Additional file 1: Figure 3A, B) was FACS sorted, and equal volumes were injected intravenously into NSG mice. Mice were monitored using in vivo BLI. **B**, **C** AML burden in individual mice of the 10 × LIC and 100 × LIC groups measured by BLI. The dotted line indicates a total flux of 5 × 10^7^ photons/s as the cut-off for evaluating positive AML engraftment. Statistical analysis was performed using Chi-squared test. Representative images of mice injected with 10 × LIC (**D**) and 100 × LIC (**E**) on day (d) 62. Mice that died in narcosis during imaging (d.i.n.) were counted as positive if the last imaging signal showed positive engraftment (p. e.) or were excluded from analysis (ex.) if not engrafted. **F** Kaplan–Meier curve of AML-491 engraftment analysed by BLI. (**G**) Kaplan–Meier curves showing survival of mice. Statistical significance was calculated using the log-rank test. *p < 0.05, **p < 0.01, not significant (ns)

Specific targeting of AML LSCs is needed to prevent relapse and enhance the rate and duration of response to therapy in patients. We hypothesized that SIRPα-αCD123 fusion antibodies would efficiently eliminate CD123^high^ CD47^high^ LSCs due to the avidity-dependent binding of the αCD123 and SIRPα moieties. Xenograft mouse models have been widely used to investigate leukaemia-initiating cells (LICs) as surrogates for LSCs [41, 42]. To evaluate the impact of HD NK cell-dependent cytotoxicity of our antibodies on LICs, we performed an in vivo engraftment assay using residual AML PDX cells that survived an ex vivo ADCC assay (Fig. 6A). We expect that LICs are killed more efficiently with SIRPα-αCD123 fusion antibodies than with αCD123 antibodies in the ex vivo ADCC assay and thus lead to reduced engraftment of AML cells. To this end, PDX cells from two AML patients were incubated with NK cells and isotype control, αCD123 or 2 × SIRPα-αCD123 (Additional file 1: Figure S3). Only the 2 × SIRPα-αCD123 fusion antibody was used as it showed superior killing of AML PDX cells (Fig. 5F). After this ADCC, surviving PDX cells were sorted and injected into NSG mice at two doses corresponding to 10 leukaemia-initiating cells (10 × LIC) or 100 × LIC. AML engraftment was analysed by in vivo BLI and peripheral blood analysis*.*

As expected, all mice that received residual cells from isotype control-treated AML-491 ADCC culture exhibited PDX cell engraftment soon after transplantation [10 × LIC: 28–49 days post injection (dpi), *n* = 5; 100 × LIC: 33 dpi, *n* = 5], whereas treatment with the αCD123 antibody slightly delayed the time to positive engraftment (10 × LIC: 62–91 dpi, *n* = 5; AML-491 100 × LIC: 49 dpi, *n* = 4) (Fig. 6B–F). Importantly, residual cells from 2 × SIRPα-αCD123 ADCC cultures exhibited a dramatically reduced engraftment capacity, with only one animal in the 10 × LIC group (114 dpi) and none in the 100 × LIC group showing positive engraftment (Fig. 6B–F). All mice with positive engraftment reached end-stage leukaemia with high BLI signals and hCD33^+^ cells in peripheral blood (Fig. 6; Additional file 1: Figure S5). AML-579 cells were injected at slightly higher doses of 14 × LIC and 140 × LIC, but the results were similar to those observed for AML-491 (Additional file 1: Figures S4 and S5).

**Fig. 7:**
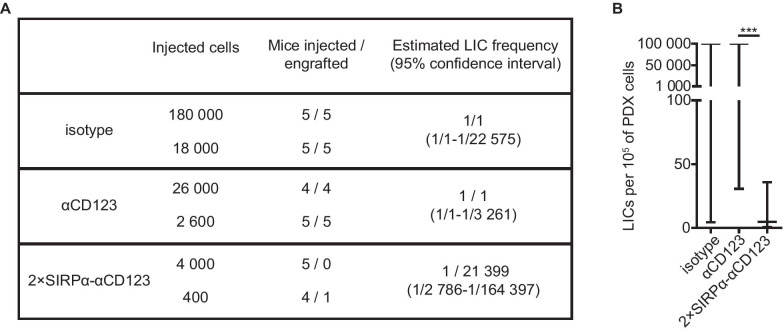
LIC frequencies of AML-491 after NK cell-mediated lysis with the 2 × SIRPα-αCD123 fusion antibody. **A** Engraftment at each injected cell dose for each antibody. Residual AML cells were counted during cell sorting before injection. **B** LIC frequencies were estimated using extreme limiting dilution analysis software [35]. Horizontal lines indicate the estimated LIC frequencies, and the bars indicate 95% confidence intervals

We used the extreme limiting dilution analysis algorithm to determine whether the nearly absent engraftment in the 2 × SIRPα-αCD123 condition was due to specific LIC targeting or a lower number of injected residual cells [35]. Even though all mice in the isotype and αCD123 treatment groups exhibited engraftment, a significant difference in the estimated LIC frequencies was detected between αCD123 and 2 × SIRPα-αCD123 for AML-491 (Fig. 7A, B; Additional file 1: Table S2). We concluded that while 2 × SIRPα-αCD123 markedly reduces the number of bulk AML cells, it targets leukemic stem cells with an even higher preference.

## Discussion

The ubiquitously expressed surface marker CD47 interacts with the SIRPα receptor to inhibit myeloid cell-mediated phagocytosis of autologous cells [4, 14, 15]. Blocking the CD47-SIRPα checkpoint as an anticancer therapy is under intense investigation since CD47 is overexpressed on AML as well as on various other cancer types [5, 10, 43]. However, ubiquitous expression of CD47 creates an antigen sink that can sequester CD47-targeting agents and reduce the effective dose. Moreover, nonspecific targeting can cause toxicities to healthy cells, as CD47 has various roles in physiological tissue homeostasis [44]. For example, the CD47 ligand SIRPγ is expressed on human T cells, and targeting CD47 with a mAb has been shown to affect human T cell responses [45].

The most serious side effects reported from CD47-targeting agents in clinical trials are anaemia and thrombocytopenia [39, 46–49]. The SIRPα-αCD123 antibodies presented here specifically bind to the AML cell line MOLM-13 in the presence of excess RBCs, in contrast to the high-affinity CD47-targeting antibody B6H12. These results agree with previous reports where similar constructs targeting CD33 and CD20 avoid the CD47 sink generated by RBCs [17, 18]. We also observed that SIRPα-αCD123 antibodies targeted PBMCs more than RBCs. The low-affinity SIRPα-dependent binding to PBMCs might, however, not lead to activation of macrophages or NK cells based on our results with SIRPα-αCD19 control molecules in experiments with MOLM-13 cells and primary AML cells. Importantly, although the SIRPα-αCD123 fusion antibodies also bind platelets, they do not induce any aggregation, unlike other CD47-targeting molecules tested herein. The underlying reason for this might be a combination of relatively low-affinity binding of the SIRPα domain to CD47 as well as different steric features of the antibody constructs.

Despite the low-affinity binding of the SIRPα domains, SIRPα-αCD123 fusion antibodies were able to induce the same or even higher phagocytosis than high affinity αCD47 either alone or in combination with αCD123. This is in line with the well-known synergy between CD47-SIRPa axis disruption and prophagocytic signals [8, 10, 50–52] and supports the rationale of combining CD47 blockade and FcγR stimulation into one molecule.

AML LSCs reside in specific niches in the bone marrow [53]. Antibodies can freely access the bone marrow through sinusoidal clefts and therefore represent a promising therapeutic strategy for targeting LSCs in their microenvironment [54]. CD33-targeting gemtuzumab ozogamicin is currently the only antibody-based therapy approved for AML [55]. Unfortunately, only some patients are likely to benefit from gemtuzumab ozogamicin [56, 57]. CD33/CD47 cotargeting has been previously preclinically investigated [18, 58]. However, bivalent mAbs against CD33 have been shown to internalize upon cross-linking, which can compromise the immune response [59, 60]. Expression of CD33 on LSCs is also associated with variability, which might affect therapeutic outcomes [20, 61]. Our results indicate that the SIRPα-αCD123 constructs are comparable to αCD33-based fusion antibodies in inducing autologous ADCP or allogenic ADCC. Interestingly, we observed much higher activation of NK cells in response to 2 × SIRPα-αCD123 than with the αCD33 analogue. Whether this was due to CD33-related internalization effects or other reasons remains to be investigated, but we consider αCD123-based constructs promising candidates next to αCD33-targeting antibodies.

Because chemorefractory LSCs build a reservoir for relapse, elimination of these cells is essential for AML treatment [1, 2]. In younger adults, a lower percentage of CD123^+^ LSCs at diagnosis is correlated with a better response to treatment and survival [62]. Similarly, in older patients who are fit for intensive chemotherapy, survival was higher in those who displayed lower levels of CD123^+^ LSCs [63]. Therefore, eliminating or reducing the numbers of CD123^+^ LSCs might lead to more durable responses and prolonged survival. We show here that compared to αCD123, SIRPα-αCD123 antibodies exhibit increased targeting efficacy of CD123^+^ CD47^+^ AML cells due to avidity-dependent binding to both antigens. Our fusion antibodies could take advantage of the high expression of both CD123 and CD47 on LSCs and effectively address this population. Indeed, we observed an extreme reduction in the engraftment of AML after an ex vivo ADCC assay with the 2 × SIRPα-αCD123 antibody, as our antibodies stimulated NK cell-mediated cytotoxic lysis of AML LSCs. The increased avidity of SIRPα-αCD123 antibodies thus provides the opportunity to preferentially target and eliminate AML LSCs.

Because of avidity-dependent binding to CD123 and CD47, SIRPα-αCD123 antibodies could further target malignant LSCs cells over healthy haematopoietic stem cells, which express low levels of CD47 and minimal amounts of CD123 [5, 19, 21, 64]. The 2 × SIRPα-αCD123 fusion antibody facilitated the most potent NK cell activation in our assays, and only this antibody was evaluated in LSC targeting experiments. To further analyse the safety and efficacy of the molecules and to determine whether the 1 ×- or 2 × SIRPα-αCD123 fusion format would be favourable in future clinical trials, assessing competitive targeting of patient-derived LSCs and healthy haematopoietic stem cells would be very pertinent.

While we are the first to combine CD123 and CD47 targeting, other therapeutic molecules have been developed against CD123 alone [65–67]. Talacotuzumab is an αCD123 antibody with a modified Fc region for enhanced ADCC [37, 68]. Unfortunately, talacotuzumab showed limited in vivo efficacy in clinical studies, which has been associated with the compromised NK cell activity in MDS and AML [69–71]. This suggests that recruiting other immune cells, such as macrophages, could stimulate a broader response to antibody-based CD123-targeting therapies. The benefit of activating macrophages in AML has been demonstrated by the αCD47 antibody magrolimab in combination with azacytidine [8]. Recent data additionally suggest that upon activation, NK cells can upregulate SIRPα, which leads to strong inhibition of cytotoxicity when interacting with CD47 on the surface of target cells [72]. An effective blockade of CD47 signalling could therefore be the reason we observed an extremely potent upregulation of CD69 on NK cells in response to 2 × SIRPα-αCD123 treatment. This was also indicated by the slight increase in the percentage of CD69^+^ cells when the αCD47 antibody Hu5F9-G4 was used. A growing body of evidence indicates that adaptive immunity, especially the activation of CD8^+^ T cells, further contributes to the effects observed in response to CD47-SIRPα inhibition [73–75]. As SIRPα-αCD123 fusion antibodies improve phagocytosis of AML patient cells compared to αCD123 while still initiating strong NK cell activation, we propose that SIRPα-αCD123 fusion antibodies stimulate a much broader immune response, including a long-lasting T-cell response.

## Conclusions

In summary, we demonstrated that SIRPα-αCD123 antibodies specifically target LSCs, mediate their efficient clearance and stimulate phagocytosis of AML while restricting CD47-related on-target off-leukaemia toxicity. These encouraging results establish SIRPα-αCD123 antibodies as a promising approach for LSC targeting for prolonged remission in AML patients. Future in vivo studies using an appropriate AML mouse model are necessary for the translation of this approach into a clinical setting.

## Supplementary Information

**Additional file 1**. Supplementary tables and figures.

## Data Availability

All data generated or analysed during this study are included in this published article [and its supplementary information files].

## Abbreviations

AML: Acute myeloid leukaemia
LSC: Leukemic stem cell
SIRPα: Signal regulatory protein alpha
FcγR: Fcγ receptor
MDS: Myelodysplastic syndrome
OR: Objective response
CR: Complete remission
NK: Natural killer
V_L_: Variable light
V_H_: And variable heavy
SDS: Sodium dodecyl sulphate
EC_50_: Half maximal effective concentration
PCR: Polymerase chain reaction
*K*_D_: Equilibrium dissociation constant
CHO: Chinese hamster ovary
PBMC: Peripheral blood mononuclear cells
PDX: Patient-derived xenograft
NSG: NOD/SCID gamma null mice
MFI: Median fluorescence intensity
RBC: Red blood cells
E:T: Effector-to-target
LIC: Leukaemia-initiating cell
BLI: Bioluminescence imaging
ANOVA: Analysis of variance
ADCP: Antibody-dependent cellular phagocytosis
ADCC: Antibody-dependent cellular cytotoxicity
HD: Healthy donor
FACS: Fluorescence-activated cell sorting
