# Supplementary material for: SIRPα-αCD123 fusion antibodies targeting CD123 in conjunction with CD47 blockade enhance the clearance of AML-initiating cells
Source: J Hematol Oncol. 2021 Sep 27;14:155. doi: 10.1186/s13045-021-01163-6 (PMC8477557; doi:10.1186/s13045-021-01163-6)
Supplement: Supplementary file 1 — Additional file 1. Supplementary tables and figures. [file 13045_2021_1163_MOESM1_ESM.docx]

**Supplementary tables and figures**

**Supplementary Table 1. Patient karyotype.**

| **Patient** | **Karyotype details** |
| --- | --- |
| 0276 | aberrant, 46,XX,t(6;11)(q27;q23) |
| 2562 | intermediate aberrant, t(15)(q22),t(17)(q21) |
| 3140 | normal |
| 3073 | normal |
| 1233 | complex aberrant, 46,X,i(Y)(p10)[3]/47,sl,+i(Y)(p10)[4]/48,sdl1,+21[8]/49,sdl2,+i(Y)(p10)[2]/46,XY[11] |
| 3826 | aberrant, 46,XY,der(7)t(7;13)(q11;q12)[19]/45,XY,-7[7]/46,XY,der(7)del(7)(p1?2)del(7)(q11)[2]/46,XY[2] |
| 2449 | aberrant, 46,XX,t(8;21)(q22;q22)[10]/46,XX[5] |
| 4169 | aberrant, 46,XY,del(9)(q?13q?22)[11]/46,XY[12] |
| 0178 | complex aberrant, 46,XX,t(16;16)(p13;q22)[6]/46,idem,der(7)(t(7;11)(p?;q23)[2]/47,idem,+9[3]/46,XX[2] |
| 3386 | normal |
| 3776 | normal |
| 3221 | normal |
| 3495 | normal |
| 0885 | normal |
| 4321 | normal |
| 6789 | normal |
| 0252 | aberrant, del(1)(q32),der(2)t(1;2)(?q32;q37) |
| 1421 | aberrant/normal, 46,XX,inv(16)(p13q22)[11]/46,XX[5] |
| 0682 | complex aberrant 45,XX,t(1;11)(p32;p11),der(9)t(9;21)(p2?4;q?),-16,der(21)t(16;21)(p13;q22)[4]/47,XX,del(2)(p1?3),del(2)(q3?1),+r(2)(?),der(9)t(9;21)(p2?4;q?),der(16)del(16)(p11)del(16)(q12),der(21)t(16;21)(p13;q22)[6] |
| 7782 | complex aberrant, 46,XY,der(11)t(9;11)(p22;q23),del(13)(q22q34)[3]/45,sl,-Y,der(9)t(Y;9)(p11;p11)[8] |
| 5964 | complex aberrant,45,X,der(X)t(X;7)(q22;p1?3),der(3)(3qter->3q26::3p26->3q21::9?->9?::3q21->3q26::3q26->3qter),der(3)t(3;?9)(q21;?),del(5)(q14),der(7)(Xqter->q?13::7p1?2->7q22::8q22->8qter),dic(8;22)(q1?;p1?)t(8;11)(p2?;?)[8]/45,X,der(X)t(X;7)(q22;p1?3),der(3)(3qter->3q26::3p26->3q21::9?->9?::3q21->3q26::3q26->3qter),der(3)t(3;?9)(q21;?),del(5)(q14),der(7)(Xqter->q?13::7p1?2->7q22::8q22->8qter),-8,ins(21;22)(q21;q11q13),add(22)(p1?)[3] |
| AML-491 | del(7)(q21) |
| AML-579 | normal |
| AML-640 | t(11;15) |
| AML-979 | normal |

**Supplementary Table 2. LIC frequencies of AML-579 cells after NK cell-mediated lysis with the** **2×SIRPα-αCD123 fusion antibody and historical controls.**

| Sample | Number of injected cells | Number of mice  injected / engrafted | Estimated LIC frequency  (95% confidence interval) |
| --- | --- | --- | --- |
| AML-579, isotype | 50 000 | 2 / 2 | 1/1 (1/1 - 1/7 800) |
|  | 5 000 | 4 / 4 |  |
| AML-579, αCD123 | 20 000 | 2 / 2 | 1/1 443 (1/424 – 1/4 908) |
|  | 2 000 | 4 / 3 |  |
| AML-579,  2×SIRPα-αCD123 | 3 000 | 2 / 2 | 1/1 674 (1/410 - 1/6 842) |
|  | 300 | 4 / 0 |  |
| Historic AML-491 (1) |  |  | 1/1 799 (1/945 - 1/3 426) |
| Historic AML-579 (1) |  |  | 1/351 (1/776-1/1 590) |

Residual AML cells were counted before injection during cell sorting and LIC frequencies were estimated using the extreme limiting dilution analysis software (2). LIC frequencies of historic untreated AML-491 and AML-579 were previously determined by Ebinger et al. (1).


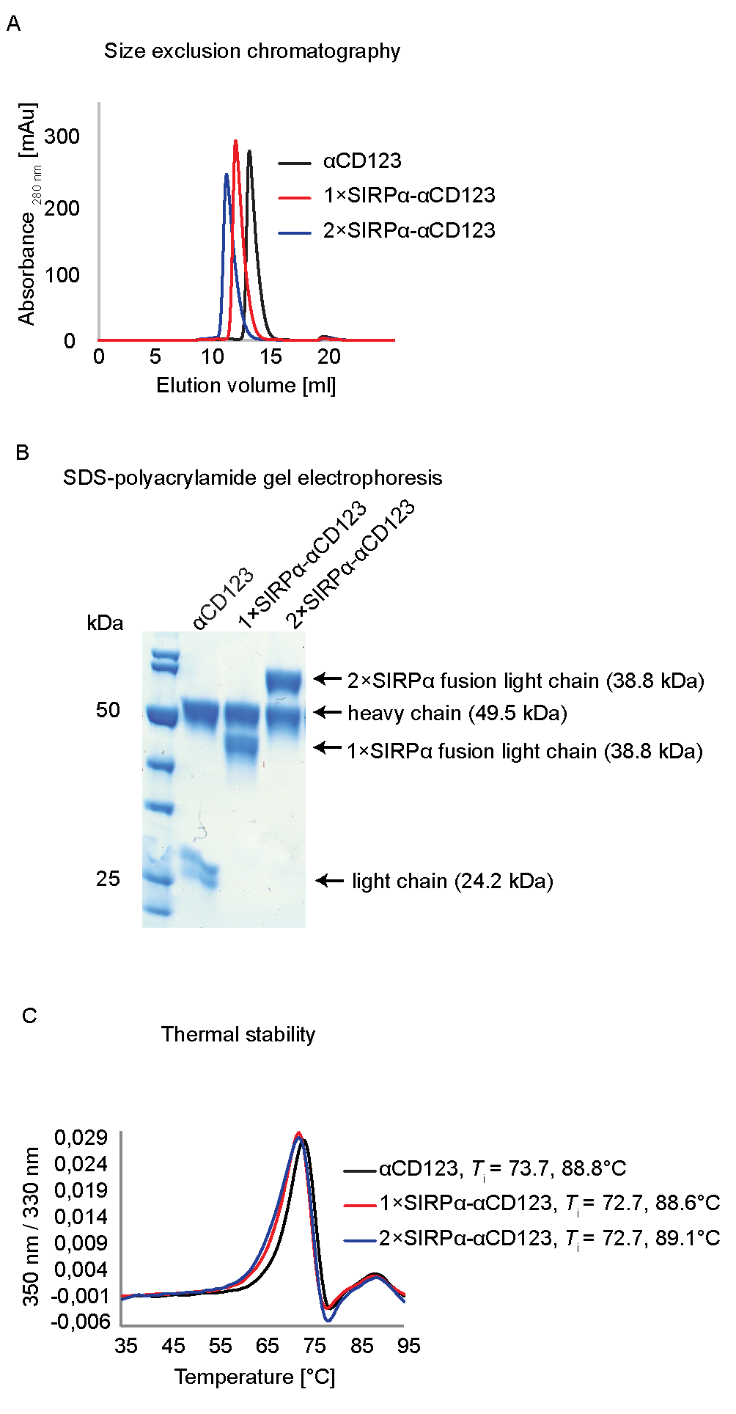


**Supplementary Figure 1. Generation and characterization of the SIRPα-αCD123 fusion antibodies.** (A) Size exclusion chromatography graphs of the antibody purification. (B) SDS-polyacrylamide gel electrophoresis of the purified antibodies under reducing conditions. (C) Thermal stability of antibodies determined by Tycho NT.6 with inflection temperatures (*T*_i_), *n* = 2 of a single experiment.

**
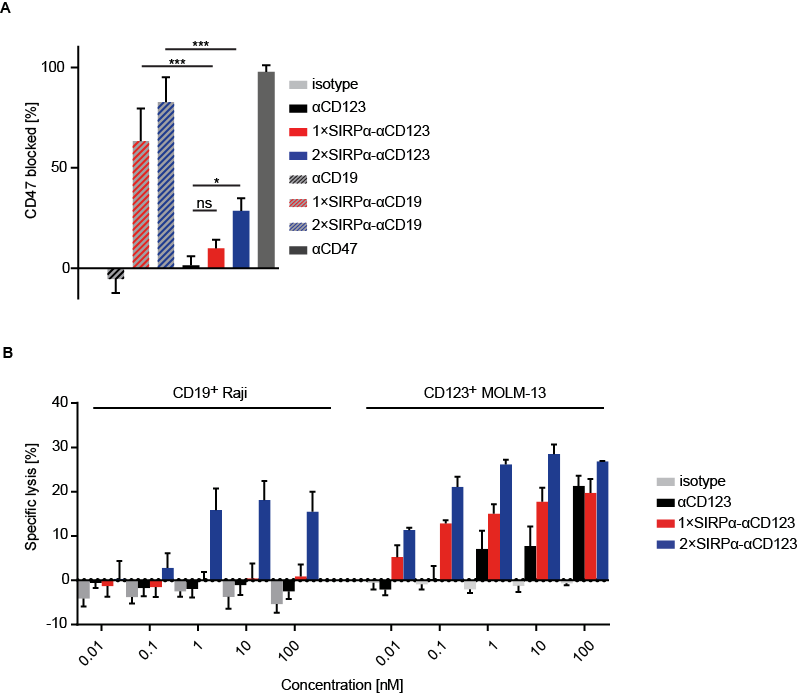
**

**Supplementary Figure 2. SIRPα-αCD123 fusion antibody effect on CD123^-^ CD19^+^ Raji cells.** (A) CD47 blockade on Raji cells with 100 nM antibodies determined by FITC αCD47 binding measured by flow cytometry. Shown are mean values ± SEM of *n* = 3 independent experiments. Statistical differences were determined by one-way ANOVA with Holm-Sidak's post-hoc test. **p* < 0.05, ****p* < 0.001, *****p* < 0.0001, not significant (ns). (B) Dose-dependent lysis of either Raji or MOLM-13 cells in a mixture of both. NK cells were incubated with MOLM-13 and Raji cell mixture for 4 h at an E:T:T ratio of 5:1:1 in the presence of serial dilutions of antibodies. Lysis was measured by Calcein AM release from the indicated cell line. Shown are mean values ± SEM, *n* = 2 different NK cell donors for MOLM-13 and *n* = 3 Raji.

**
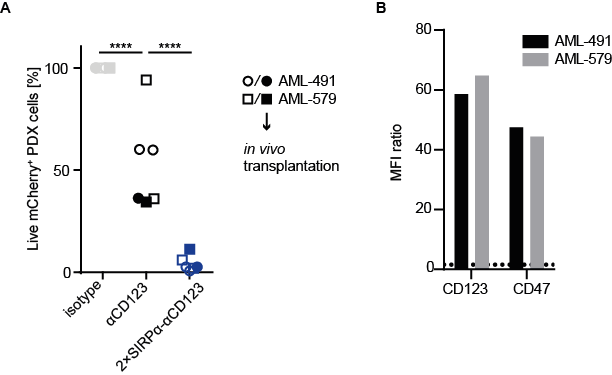
**

**Supplementary Figure 3. Analysis of AML PDX cells before the transplantation study.** (A) NK cell-mediated lysis of AML-491 and AML-579 cells at 100 nM antibody concentration after 20 h at an E:T ratio of 5:1 measured by flow cytometry. For both AML-491 and AML-579 cell, *n* = 3 assays were performed with different HD-derived NK cells. Filled shapes indicate samples that were used for the transplantation study. NK cells from different donors were used with AML-491 and AML-579 in the ADCC experiment for transplantation. Statistical differences were determined by one-way ANOVA with Holm-Sidak's post-hoc test. *****p* < 0.0001. (B) Samples that were used in the transplantation study were analyzed by flow cytometry to detect the expression of CD123 and, *n* = 1.

**
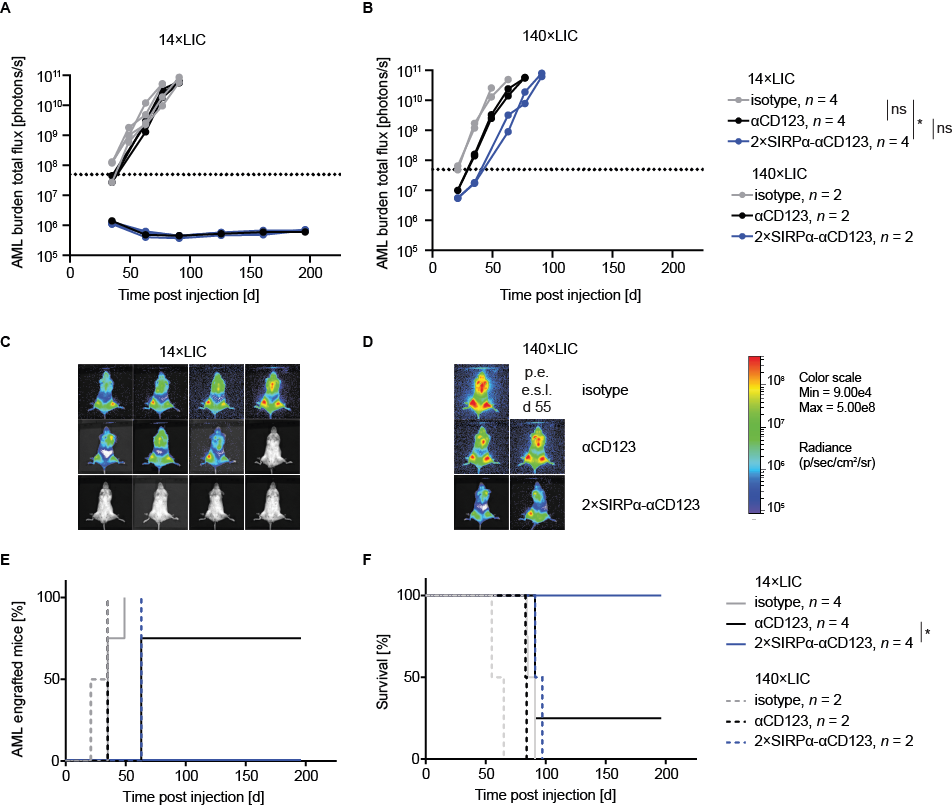
**

**Supplementary Figure 4**. **SIRPα-αCD123 pre-treatment prevents outgrowth of AML-579** **in an *in vivo* engraftment experiment.** (A, B) AML burden in individual mice of the 14×LIC (A) and 140×LIC (B) groups measured by BLI. Dotted line indicates total flux of 5×10^7^ photons/s as cut-off for evaluating positive AML engraftment. Representative imaging pictures of mice injected with 14×LIC (C) and 140×LIC (D) on day (d) 63. Positive engraftment (p. e.), end stage leukemia (e. s. l.). (E) Kaplan–Meier curve of the AML-579 engraftment analyzed by BLI. (F) Kaplan–Meier curves showing survival of mice. Statistical significance was calculated with the log-rank test. **p* < 0.05.


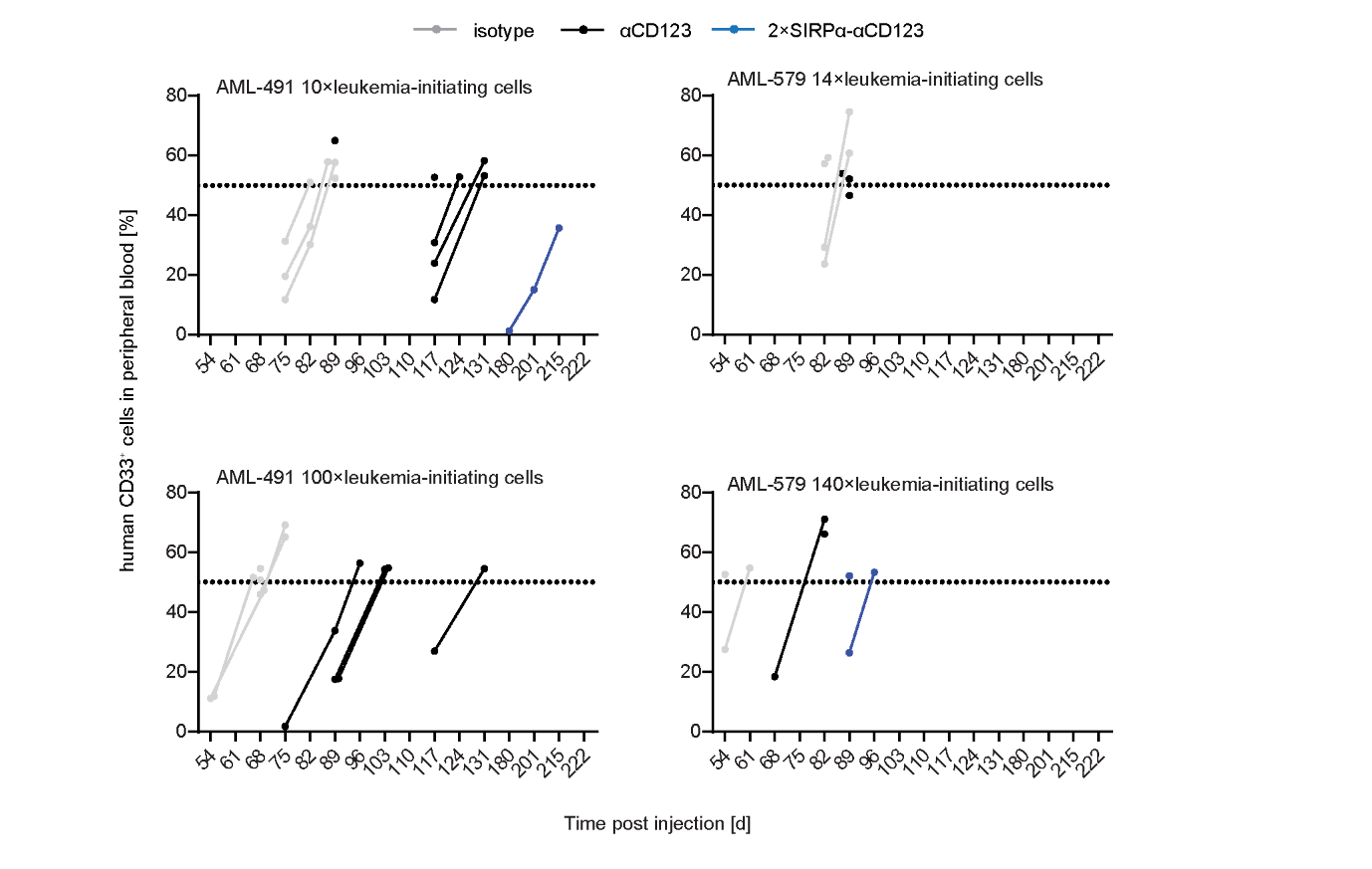


**Supplementary Figure 5.** **High levels of human AML cells in positively engrafted mice.** Percentage of human CD33^+^ cells measured from peripheral blood of the mice after injection of the AML PDX cells. Dotted line indicates > 50% as one of the indicators for end stage leukemia. Mice showing no positive signal in highly sensitive bioluminescence imaging were not analyzed. Day (d).

**References:**

1. Ebinger S, Zeller C, Carlet M, Senft D, Bagnoli JW, Liu WH, et al. Plasticity in growth behavior of patients' acute myeloid leukemia stem cells growing in mice. Haematologica. 2020.

2. Hu Y, Smyth GK. ELDA: extreme limiting dilution analysis for comparing depleted and enriched populations in stem cell and other assays. J Immunol Methods. 2009;347(1-2):70-8.
